# Graph Independent Component Analysis Reveals Repertoires of Intrinsic Network Components in the Human Brain

**DOI:** 10.1371/journal.pone.0082873

**Published:** 2014-01-07

**Authors:** Bumhee Park, Dae-Shik Kim, Hae-Jeong Park

**Affiliations:** 1 Brain Korea 21 Project for Medical Science, Yonsei University College of Medicine, Seoul, Republic of Korea; 2 Department of Nuclear Medicine and Severance Biomedical Science Institute, Yonsei University College of Medicine, Seoul, Republic of Korea; 3 Department of Electrical Engineering, Korea Advanced Institute of Science and Technology, Daejeon, Republic of Korea; National Research & Technology Council, Argentina

## Abstract

Does each cognitive task elicit a new cognitive network each time in the brain? Recent data suggest that pre-existing repertoires of a much smaller number of canonical network components are selectively and dynamically used to compute new cognitive tasks. To this end, we propose a novel method (graph-ICA) that seeks to extract these canonical network components from a limited number of resting state spontaneous networks. Graph-ICA decomposes a weighted mixture of source edge-sharing subnetworks with different weighted edges by applying an independent component analysis on cross-sectional brain networks represented as graphs. We evaluated the plausibility in our simulation study and identified 49 intrinsic subnetworks by applying it in the resting state fMRI data. Using the derived subnetwork repertories, we decomposed brain networks during specific tasks including motor activity, working memory exercises, and verb generation, and identified subnetworks associated with performance on these tasks. We also analyzed sex differences in utilization of subnetworks, which was useful in characterizing group networks. These results suggest that this method can effectively be utilized to identify task-specific as well as sex-specific functional subnetworks. Moreover, graph-ICA can provide more direct information on the edge weights among brain regions working together as a network, which cannot be directly obtained through voxel-level spatial ICA.

## Introduction

Decades of neuroimaging studies have demonstrated that cognition is co-localized with cyto and/or myeloarchitectonically distinct brain areas. Yet, more recent data suggest this structure-function relationship to be highly complex such that a single cognitive function can recruit multiple distributed local clusters of neurons [1], [2]. Furthermore, diverse brain states and functions appear to be encoded by altering connectivity among distributed neuronal clusters [3]-[5]. The importance of these distributed interactions (i.e., a network) in constructing diverse cognitions is widely acknowledged in the field of systems neuroscience.

Despite a large amount of growth in phenomenological data supporting this network perspective on cognition, the mechanism behind how the brain formulates highly diverse brain processes or characterizes various individuals has not been sufficiently studied. Given that there is a potentially infinite number of different cognitive processes, does the brain generate new networks each time it computes a new cognitive process? Recent data suggest, alternatively, that pre-existing repertoires of a much smaller number of canonical network components are selectively and dynamically recruited for various cognitions [6]. To this end, relatively well-defined network components such as working memory circuits, motor circuits, and language circuits may simply be members, or mixtures of members, of these repertoires of functional network components.

The primary aim of this study was to identify independent cognitive network components from limited sets of neuroimaging data. Instead of relying on task-specific data which are impractical to cover whole brain processes, we focused on recent findings that the pool of cognitive network components are embedded in spontaneous activity [7], independent of specific cognitive tasks, in the fashion of slow fluctuations in synchrony of distributed regions during the resting state [8].

**Figure 1:**
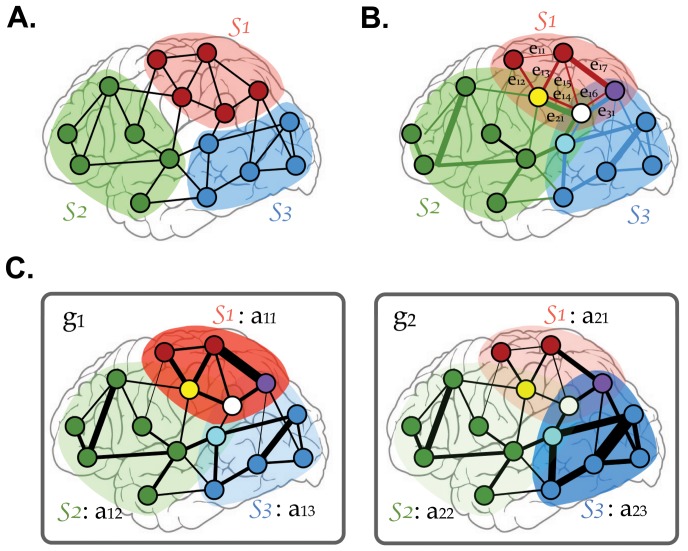
Motivation for use of graph-ICA. The graph-ICA is to decompose intrinsic subnetworks based on the neurocognitive network model with two assumptions; 1) A single edge (i.e., functional connection between two regions) can be engaged in multiple cognitive functions and can be part of multiple functional subnetworks with different weights (i.e., connectivity) (**B**), rather than a part of only a single subnetwork (**A**); 2) Whole brain networks (i.e., graphs) can be composed of independent canonical subnetworks. Each individual recruits different subnetworks with different strengths of their involvements (**C**). The usage strengths of subnetworks can be used to identify task-specific subnetworks or group-specific subnetworks. S1, S2, and S3: three subnetworks; e_ij_: j-th edge in the i-th subnetwork; g_1_ and g_2_: two exemplary whole brain networks from two individuals; a_ij_: the usage strength of j-th subnetwork in the i-th graph.

**Figure 2:**
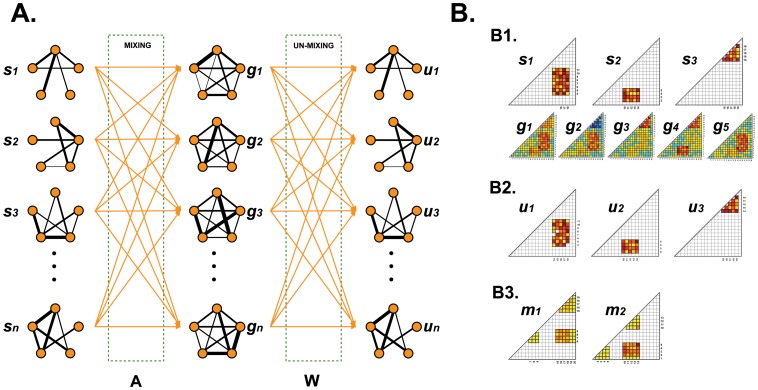
Concept and simulation results of graph-ICA. **A.** **Concept**: An individual whole brain network (**g** *_i_*) can be expressed as a weighted sum of independent source brain subnetworks (***s*** *_i_*; *i = 1,..,n*) by a mixing matrix (A). The purpose of graph-ICA is to estimate an unmixing matrix (W) and subsequently to estimate source independent brain subnetworks (***u*** *_i_*; *i = 1,..,n*) from cross-sectional whole brain networks (**g** *_i_*; *i = 1,..,n*). **B. Simulation:** We generated three original independent subnetworks (***s*** *_i_*; *i = 1,..,3*). Five artificial whole brain networks (**g** *_i_*; *i = 1,..,5*) were generated by mixing three independent original subnetworks (***s*** *_i_*) with different weights and background noise (**B1**). From whole brain networks, graph-ICA (**B2**) estimated original subnetworks better than modularity optimization (**B3**).

To identify intrinsic cognitive network components from spontaneous activity, we proposed a subnetwork decomposition method from multitudes of whole brain networks, with an assumption that repertoires of intrinsic subnetworks constitute individual brain networks with different strength combinations (Figure 1). We identified intrinsic functional subnetworks of the human brain by applying independent component analysis (ICA) [9] to a group of brain networks in the form of graphs (graph-ICA) (Figure 2). Using derived subnetwork repertoires, we decomposed brain networks during specific tasks including motor activity, working memory exercises, and verb generation, and identified subnetworks associated with performance on these tasks. We also analyzed sex differences in utilization of subnetworks, which was useful in characterizing group networks.

## Materials and Methods

### Graph-ICA concepts

Graph-ICA is a type of cross-sectional ICA that decomposes measured graphs into common source graphs (Figure 2A). We denote a graph (i.e. an adjacency matrix) with L nodes from a resting state fMRI of the *i*-th brain, a vector, **g**_i,_ with K = L(L-1)/2 edges for elements. We assumed that *N* independent network components (IC), **s**_j_, *j* = 1, …, *N*, exist in the human brain. *M* graphs from M brains, i.e., **g**_i,_ i = 1, …, M, were concatenated to a matrix **g**, and were modeled by weighted mixing of independent component matrix **s** with a mixing matrix **A**, as shown below.



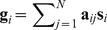
(1)where the weight *a*_ij_ is the element of **A** indicating the contribution of (graph) source **s**_j_ to compose **g**_i_. This can be rewritten as:



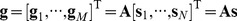
(1)


The matrix sizes of **g**, **s,** and **A** were (*M* x *K*), (*N* x *K*), and (*M* x *N*). For this study, we assumed the number of ICs (*N*) equaled the number of graphs (*M*), i.e., *N*  =  *M*  =  104, since we do not have a clear *a priori* knowledge on the number of ICs. The mixing matrix **A** can be estimated by an ICA algorithm, which maximizes mutual independence between estimated functional components [9].

### Graph-ICA: A simulation study

We applied graph-ICA to the simulated data. We generated five artificial graphs by mixing three source graphs with background noise. The three source graphs consisted of two overlapping and one non-overlapping (spatially) independent graphs (Figure 2B1). We considered 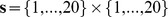 to be a total connectivity set. Three source graphs were designed to be connected only within 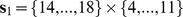, 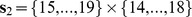, and 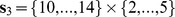. This setting satisfies 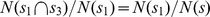 (as a spatial independence condition), where the notation N(s) represents the number of nodes in **s**. Connectivity of source graphs within existing connections were assigned for samples of uniform distribution, *U*(0.25,1). We artificially generated five graphs by summing differentially weighted three source graphs and background Gaussian noise (a contrast-to-noise ratio of 0.5, 0.75, 1, 1.5, and 2 for three sources). We applied graph-ICA to these five generated data sets using the Infomax algorithm [9] to extract ICs corresponding to three source graphs.

### Subjects

Resting state fMRI data from 104 healthy, right-handed participants (48 males and 56 females, mean age: 23 ± 6 years, age range: 10–35 years) were used in this study. For task-specific functional data, we acquired fMRI scans from 5 healthy participants during which they performed a motor task, an n-back task, and a verb-generation task. Handedness was assessed using a Korean version of the Annett handedness questionnaire [10]. None of the participants had a history of neurological illness or psychiatric disorders. All participants gave written informed consent for participation according to the Declaration of Helsinki (BMJ 1991; 302: 1194) and this study was approved by the Severance Institutional Review Board (IRB).

### Data acquisition, image processing, and construction of whole brain networks

All participants underwent fMRI scanning using a 3.0 Tesla MRI scanner (Philips Achieva, Philips Medical System, Best, The Netherlands) to obtain T2* weighted single shot echo planar imaging (EPI) axial scans with the following parameters: voxel size, 2.75×2.75×4.5 mm^3^; slice number, 29 (interleaved); matrix, 80×80; slice thickness, 4.5 mm; repetition time (TR), 2000ms; echo time (TE), 30ms; and field of view, 209×220 mm^2^. To facilitate subsequent spatial normalization, we also obtained a high resolution T1-weighted MRI volume data set for each subject using a three-dimensional T1-TFE sequence configured with the following acquisition parameters: voxel size, 0.859×0.859×1.2 mm^3^; TR, 9.6ms; and TE, 4.6ms. Foam pads were used to reduce head motion during EPI data acquisition.

For resting-state fMRI data, we acquired functional scans while participants lay resting with their eyes closed without focusing on any specific thoughts or sleeping. This was evaluated by a questionnaire that was completed after scanning. Scanning consisted of 165 volumes per participant, which took 330 seconds. 165 scans for a run are known to be sufficient to detect low frequency clustered fluctuations in resting state fMRI [11] and to reliably evaluate resting state functional connectivity [12].

We conducted pre-processing for resting state fMRI using statistical parametric mapping (SPM8, Wellcome Department of Cognitive Neurology, London, UK) [13]. This process included correction for acquisition time delay between slices and correction for head motion by realigning all consecutive volumes to the first image of the session. We discarded the first 5 scans in order to minimize stability issues and used the 160 EPI data for analysis. The realigned images were co-registered with T1-weighted images, which were then used to spatially normalize the functional data into a template using nonlinear transformation. Finally, we spatially smoothed all normalized images using a 4 mm full-width half-maximum Gaussian kernel.

To obtain individual whole brain networks, we calculated an interregional correlation map (adjacency matrix) of each mean fMRI time series between 90 cortical regions as defined by automated anatomical labeling [14].

Correlation coefficients between the mean time series of two regions were calculated after band-pass filtering (0.009–0.08 Hz) and regressing out effects of rigid motion and global signal changes in white matter, cerebrospinal fluid, and whole brain [15].

### Identification of subnetworks using graph-ICA of whole brain networks

For each participant, only upper diagonal elements of the adjacency matrix were used for principal component analysis (PCA) and graph-ICA since the adjacency matrix is symmetric in this study. To reduce redundancy in graph data from the 104 participants, 75 principal components were chosen according to 90% explained variance in PCA. For the reduced 75 principal components, we conducted ICA using the Infomax algorithm [9] 100 times. To identify reproducible ICs across 100 different trials, we applied the RAICAR (ranking and averaging independent component analysis by reproducibility) algorithm [16], which finds maximally correlated ICs across different trials and ranks them by averaging their correlations. RAICAR automatically grouped similar ICs across all trials and calculated mean correlations of grouped ICs. Of the 75 groups, we selected 49 whose mean correlations were greater than 0.3 (z-score = 19.58, p<10^−16^). Finally, the ICs were normalized to z-scores with a threshold of z>3. These resultant graph-ICs are termed independent subnetworks in this study.

These subnetworks were compared with subnetworks identified by weighted modularity optimization (Text S1). Prior to modularity optimization, we conducted Fisher’s r-to-z transformations of inter-regional functional connectivity among 90 cortical regions for each subject and averaged them. Resulting modules (subnetworks) were normalized z-scores in spatial dimension with a threshold of z>3.

To show the validity of subnetworks derived using graph-ICA, we evaluated the subnetworks based on results of conventional spatial ICA. In this evaluation, we sampled a subgroup (N = 44) from the whole 104 subjects according to their ages (21–25 years, mean age = 23). This makes it practical to conduct voxel-wise spatial ICA. According to previous studies [17], [18], it is generally acceptable to decompose the whole brain signals into around 70 independent sources without significantly over-fitting data. Since time series data (160 scans) for an individual were temporally redundant, we reduced the dimension of individual time series to 16 by using PCA according to a threshold of explained variance of 80%. However, total principal components in temporal space (total 16×44 = 704) are still higher than the number of expected source numbers, i.e., 70. We again reduced the dimension of these components to 70 by using PCA before applying ICA. Therefore, information within 44 subjects may represent well 104 subjects without loss of generality.

We subsequently conducted spatial ICA for these 70 principal components. Among these, 11 spatially independent components associated with the occipital lobes were selected. To estimate individual mixing matrices from the 11 group ICs, we applied a dual-regression approach [19]. Finally, we calculated correlations among columns of individual mixing matrices and conducted Fisher’s r-to-z transformations for each correlation coefficient and one sample t-test.

### Characterization of task-specific and group-specific subnetworks

To evaluate the subnetworks associated with specific cognitive functions, we projected whole brain networks during three cognitive tasks onto the independent subnetworks identified using the resting state whole brain networks. The motor and cognitive task procedures were as follows:

**Motor task.** For 7 subjects, the fMRI session included 6 alternating blocks of 3 motor activation task blocks (30 seconds per block), and 3 resting blocks (30 seconds per block). During the motor activation task, subjects were instructed to continuously move their left foot while minimizing movement of the right foot and body. During resting blocks, subjects were instructed not to move the right foot or other body parts.

**N-back task.** We used 2-back tasks as experimental tasks and 0-back tasks as control tasks reflecting verbal working memory. For 0-back tasks, subjects were instructed to respond every time a target stimulus was presented. For 2-back tasks, subjects were instructed to respond whenever a pre-indicated stimulus that had been presented was presented again after one intervening stimulus. The stimuli were auditory, and included three Korean nouns (socks, pencil, and plate). The block-designed experiment was conducted according to the stimulus condition. The sequence of blocks was composed of three alternating sets of 0-back and 2-back tasks. The order of the stimulus conditions was counter-balanced across subjects. Ten stimuli were presented for 25s each in a 0-back task block, whereas 14 stimuli were presented for 35s each in a 2-back task block. Each block began with instructions for the subsequent task.

**Verb-generation task.** For 5 subjects, the fMRI session included 10 alternating blocks of 5 verb-generation task blocks (30s per block), and 3 resting blocks (30s per block). During the verb-generation task, subjects were instructed to continuously generate relevant verbs for visually presented texts.

All data were preprocessed in the same manner as the resting-state fMRI data. To evaluate task-dependent network structures during block-designed tasks, we split fMRI data into task and baseline blocks (movement versus resting, 2-back versus 0-back, and verb-generation versus resting) and concatenated them separately. Then, we calculated interregional correlation maps (task or baseline adjacency matrices) for separately concatenated time series. Each adjacency matrix in the graph was regressed on column spaces (weighted edge vectors) of 49 subnetworks derived from 104 resting-state networks with intercept. The regression coefficients (beta; usage-strength) were compared between task and baseline using a paired t-test.

We also compared usage-strengths between males and females for each subnetwork after linearly projecting individual adjacency matrices onto identified subnetworks. Each correlation map was regressed on spaces of 49 subnetworks with intercept. The regression coefficients were compared between males and females by 10,000x random permutation tests.

## Results

### Simulation results and comparisons of graph-ICA and voxel-level spatial ICA

In evaluating the validity of using graph-ICA for decomposing subnetworks, a simulation using a weighted mixture of original edge-sharing subnetworks showed advantages of graph-ICA over modularity optimization [20] (Figure 2B). Graph-ICA successfully determined the distribution of weighted edges of the original subnetworks. The performance evaluation according to contrast-to-noise ratio (see Figure S1) shows that contrast-to-noise ratio over than 1 is acceptable to reliably decompose initial sources.

**Figure 3:**
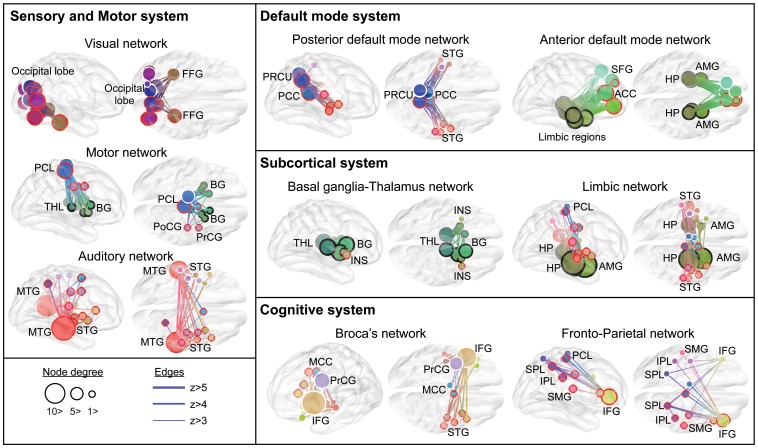
Representative functional subnetworks identified by graph-ICA. Each group IC was thresholded by z = 3. The line width indicates the weight of the edge. The size of a circle indicates node degree at the node. ACC: anterior cingulate cortex; AMG: amygdala; BG: basal ganglia; FFG: Fusiform gyrus; HP: hippocampus; IFG: inferior frontal gyrus; INS: insula; IPL: inferior parietal lobule; MCC: middle cingulate cortex; MTG: middle temporal gyrus; PCC: posterior cingulate cortex; PCL: paracentral lobule; PoCG: postcentral gyrus; PrCG: precentral gyrus; PRCU: precuneus; SFG: superior frontal gyrus; SMG: supramarginal gyrus; SPL: superior parietal lobule; STG: superior temporal gyrus; THL: thalamus.

**Figure 4:**
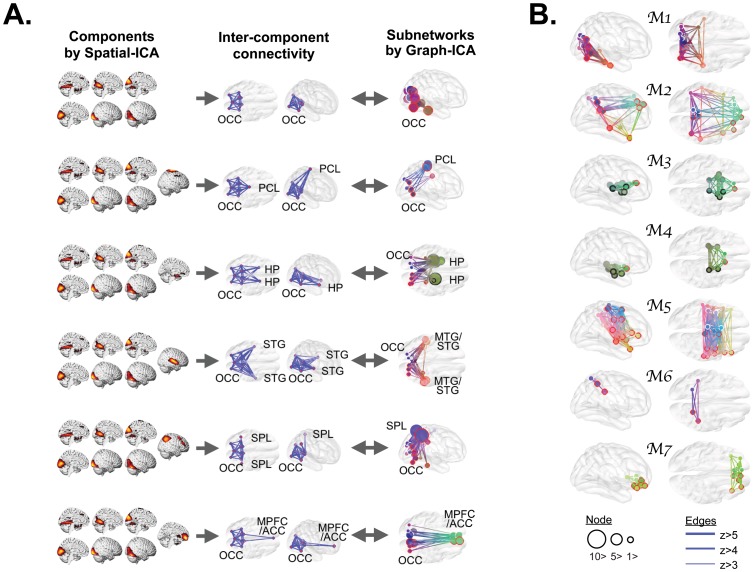
Comparisons among graph-ICA, voxel-level spatial ICA, and modularity optimization. A. Comparisons with voxel-level spatial ICA: We compared subnetworks based in the occipital cortex derived using graph-ICA with inter-component networks derived using spatial ICA. Inter-component networks were obtained by calculating correlation coefficient between weights of ICs derived using voxel-level spatial ICA. Subnetworks derived using graph-ICA had high correspondences with networks composed of spatial ICs. B. Functional subnetworks estimated by modularity optimization: These subnetworks were also compared with subnetworks identified using graph-ICA. The line width indicates the weight of the edge. The size of a circle indicates node degree at the node. ACC: anterior cingulate cortex, HP: hippocampus, MPFC: medial prefrontal cortex, MTG: middle temporal gyrus, OCC: occipital lobe, PCL: paracentral lobule, SPL: superior parietal lobule, STG: superior temporal gyrus.

To relate graph-ICA results (Figure 3), which will be illustrated in the next section, with voxel-level spatial ICA, 11 spatially independent components associated with the occipital lobes were chosen to form functional networks among them. Subnetworks estimated by graph-ICA were similar to the networks defined with inter-component connectivity using spatial ICA as presented in the Figure 4A.

### Functional subnetworks uncovered by graph-ICA

A total of 104 individual brain networks containing 90 cortical nodes and their connectivities were decomposed into 49 intrinsic functional subnetworks. Graph-ICA differentiated sensory, motor, default mode, subcortical, and higher cognitive subnetwork systems from whole brain networks. Figure 3 describes representative functional subnetworks identified by graph-ICA. Others subnetworks are listed in Figure S2. These subnetworks are comparable to network modules identified using modularity optimization (see Figure 4B).

The vision subnetwork (IC1) is composed of dense connections among regions of the occipital lobe. The auditory subnetwork (IC30) is primarily based in the middle temporal gyrus that acts as a hub of connectivity for most temporal regions. The motor subnetwork (IC19) based in the paracentral lobule consists of connections between the pre- and postcentral gyrus and the thalamus/basal ganglia. We also identified two default mode subnetworks including posterior and anterior subnetworks. The posterior default mode subnetwork (IC34) is mainly based in the posterior cingulate cortex/precuneus in connection with the temporal regions bilaterally. The anterior default mode subnetwork (IC3), on the other hand, is primarily located within the anterior cingulate cortex, medial superior frontal gyrus, and medial orbitofrontal cortex in connection with the limbic regions bilaterally.

**Figure 5:**
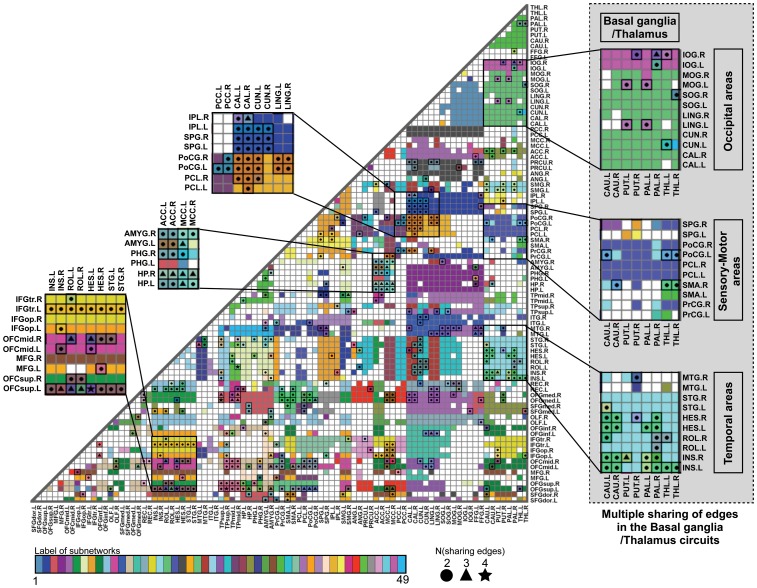
Subnetworks in the adjacency matrix and shared edges. Each subnetwork is composed of edges with a same color. Edges shared by multiple subnetworks were color-coded by averaging colors of multiple subnetworks. Circles (2), triangles (3), and star shapes (4) within cells represent the number of subnetworks sharing that particular edge. ACC: anterior cingulate cortex, AMG: amygdala, CAL: calcarine, CAU: caudate, CUN: cuneus, HES: heschl gyrus, HP: hippocampus, IFGtr: triangular part of inferior frontal gyrus, IFGop: opercular part of inferior frontal gyrus, INS: insula, IOG: inferior occipital gyrus, IPL: inferior parietal lobule, LING: lingual gyrus, MCC: middle cingulate cortex, MFG: middle frontal gyrus, MOG: middle occipital gyrus, MTG: middle temporal gyrus, OFCmid: middle orbitofrontal cortex, OFCsup: superior orbitofrontal cortex, PAL: pallidum, PCC: posterior cingulate cortex, PCL: paracentral lobule, PHG: parahippocampal gyrus, PoCG: postcentral gyrus, PrCG: precentral gyrus, PUT: putamen, ROL: rolandic fissure, SMA: supplementary motor area, SOG: superior occipital gyrus, SPL: superior parietal lobule, STG: superior temporal gyrus, THL: thalamus. L: left, R: right.

We identified two typical subcortical network systems: a dense subnetwork connecting the basal ganglia and thalamus (IC33), and a subnetwork based in the limbic regions (IC7). We also found two representative higher cognitive subnetworks including a subnetwork based in Broca’s area (IC37) and another in the fronto-parietal subnetwork (IC35) (Figure 3). Additional diverse cognitive subnetworks are listed as the adjacency matrix in the Figure 5.

Some subnetworks share single or multiple edges, as shown in Figure 5. Edges may be shared by up to 4 subnetworks. Figure 5 shows an example of shared edges/nodes including those in the basal ganglia and thalamus.

### Task-specific subnetworks and sex-specific subnetworks

The differential involvement of each subnetwork was evaluated during motor and cognitive tasks (n-back and verb-generation tasks) by linearly projecting the individual adjacency matrices onto identified subnetworks derived from 104 resting state networks. The individual linear weights represent the strength of involvement of the subnetworks during the tasks, or the network usage-strengths.

**Figure 6:**
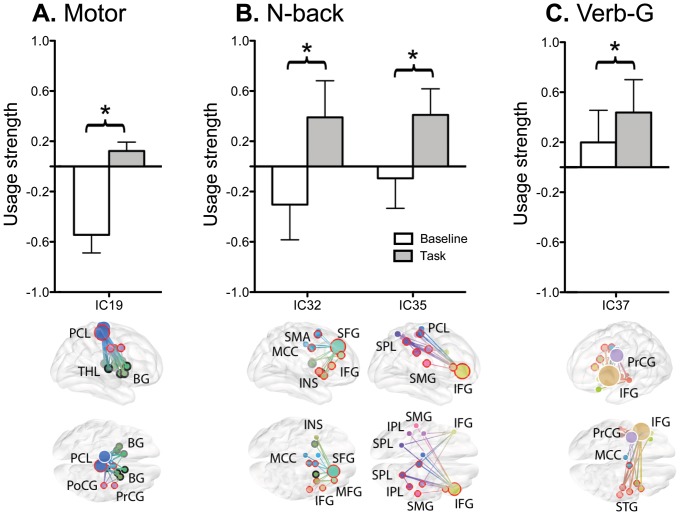
Involvement of specific functional subnetworks in cognitive tasks. **A.** Motor performance recruited more the paracentral lobule network (IC19). **B.** N-back task recruited more the fronto-parietal network (IC35) and the subnetwork cored at the right superior frontal gyrus (IC32). **C.** The verb-generation task recruited more a subnetwork cored at the left inferior frontal gyrus (IC37). * : p<0.05 after Bonferroni correction. BG: basal ganglia; IFG: inferior frontal gyrus; INS: insula; IPL: inferior parietal lobule; MCC: middle cingulate cortex; MFG: middle fronta gyrus; PCL: paracentral lobule; PoCG: postcentral gyrus; PrCG: precentral gyrus; SFG: superior frontal gyrus; SMA: supplementary motor area; SMG: supramarginal gyrus; SPL: superior parietal lobule; STG: superior temporal gyrus; THL: thalamus.

Statistical comparisons of usage-strengths for each subnetwork showed that motor performance recruited a significant portion of the paracentral lobule network (IC19, p = 0.001, Bonferroni-corrected), which has weighted connections with the basal ganglia, thalamus, right postcentral gyrus, and right precentral gyrus (Figure 6A). The 2-back task recruited significantly more of the fronto-parietal network (IC35, p = 0.0005, Bonferroni-corrected) as well as the subnetwork based in the right superior frontal gyrus (IC32, p = 0.0006, Bonferroni-corrected) compared to the 0-back task (Figure 6B). Additionally, a network based in the left inferior frontal gyrus (IC37) was highly involved in the verb-generation task (p = 0.0005, Bonferroni-corrected) (Figure 6C).

**Figure 7:**
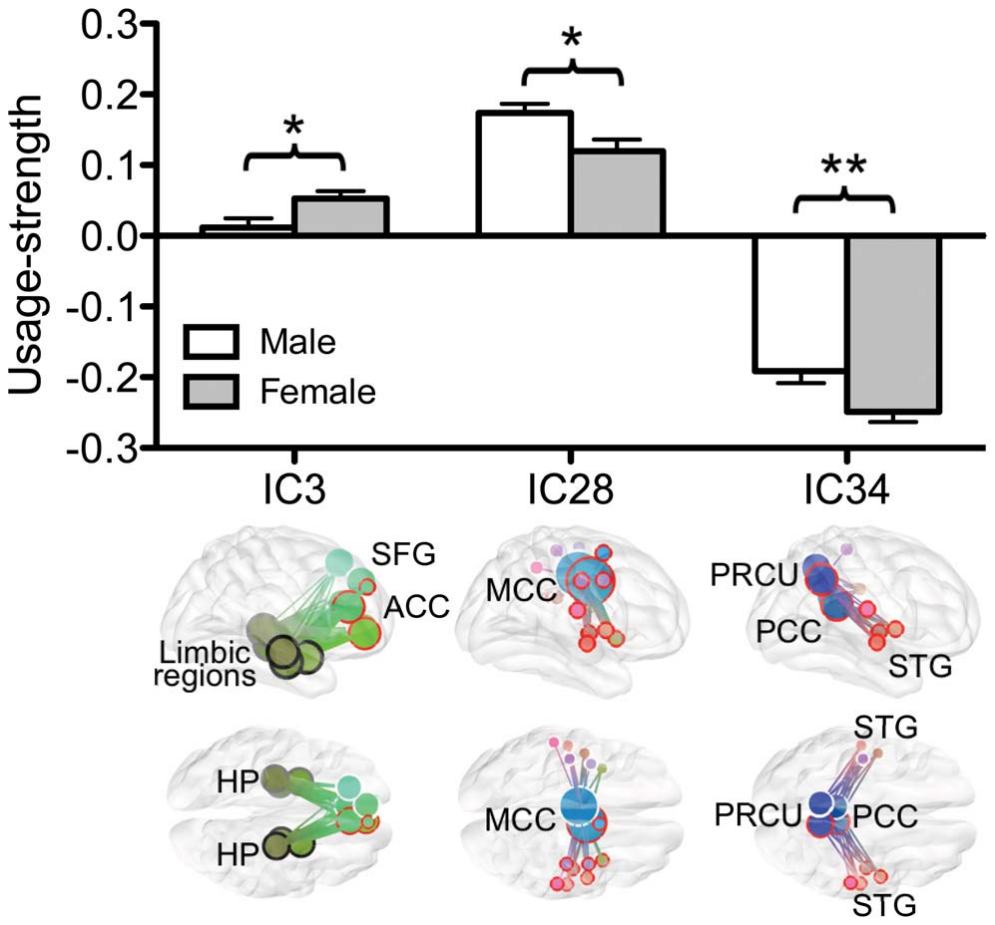
Group-level comparisons of functional subnetworks using usage weights: sex-effects. Sex differences were found in comparing usage-strengths of three subnetworks. These include the networks cored at the posterior cingulate cortex/precuneus (IC34), the middle cingulate cortex (IC28), and connecting the medial prefrontal and limbic regions (IC3). * : p<0.05, and ** : p<0.01. ACC: anterior cingulate cortex; HP: hippocampus; MCC: middle cingulate cortex; PCC: posterior cingulate cortex; PRCU: precuneus; SFG: superior frontal gyrus; STG: superior temporal gyrus.

When sex differences were evaluated, usage-strengths of the networks based in the posterior cingulate cortex/precuneus (IC34) (p = 0.004, 10,000x random permutation test) and the middle cingulate cortex (IC28) (p = 0.007, 10,000x random permutation test) were greater in males than in females (Figure 7). On the other hand, the usage-strength of the network connecting the medial prefrontal and limbic regions (IC3) was less in males compared to females (p = 0.008, 10,000x random permutation test) (Figure 7).

## Discussion

In order to identify independent cognitive network components from limited sets of neuroimaging data, we extracted 49 independent subnetworks derived from a group of whole brain networks embedded in the resting state activities. These included sensorimotor, default mode, subcortical, and higher cognitive system subnetworks.

Many attempts have been made to identify functional constructs. Mesulam [6] proposed that the human brain has five fundamental neurocognitive networks. These include a spatial attention network based in the posterior parietal cortex and frontal eye fields, a language network based in the middle temporal gyrus as well as Wernicke’s and Broca’s areas, an explicit memory network based in the hippocampal-entorhinal complex and inferior parietal cortex, a face-object recognition network based in the ventral temporal cortex and anterior temporal lobe, and a working memory-executive function network based in the dorsolateral prefrontal and inferior parietal cortices. Most of these neurocognitive networks have been identified by functional connectivity studies using resting state fMRI [7].

It has also been suggested that three particular core networks detected during resting state activity participate in higher cognitive functions; that is, the central-executive network based in the dorsolateral prefrontal cortex and posterior parietal cortex, the salience network based in the anterior insula adjoining the fronto-insular cortex and anterior cingulate cortex, and the default mode network based in the posterior cingulate cortex, medial prefrontal cortex, medial temporal lobe, and angular gyrus [21]–[23].

Subnetworks found in this study have considerable overlap with spatially independent functional patterns introduced in previous studies [6], [7], [21]–[23], though the current subnetworks were decomposed directly from multitudes of whole brain networks rather than from time series data. In previous spatial ICA studies, network information was indirectly driven by referring to the spatial co-distribution (or co-occurrence) within a component. In other words, one or more clusters within an independent component can be considered to be ‘functionally connected’ as they covary together. However, spatial ICA does not provide information on the connectivity (i.e., strength of connection) among clusters within the component. Since the current method focuses on edges rather than nodal activities, the association between nodes can be more clearly demonstrated than in previous spatial ICA studies.

In the current study, subnetworks were built on a pool of intrinsic edges covering the whole brain network. Several subnetworks were assembled utilizing common edges. For example, some edges in the basal ganglia/thalamus circuits were shared not only in subnetwork IC33, but also in subnetworks including the occipital regions (IC18, IC60), temporal regions (IC17), frontal regions (IC9, IC16, IC29), and motor regions (IC19). Certain edges in the posterior cingulate cortex/precuneus circuits were also shared with various subnetworks such as the posterior default mode subnetwork (IC34), a subnetwork consisting of Broca’s area (right) (IC58), a subnetwork consisting of the motor regions (IC27), and a subnetwork that included the occipital regions (IC20).

In this model, what make each subnetwork unique is not exclusive usage of certain edges, but rather the weighted usage of those edges. Although a functional edge between two nodes may largely be mediated by anatomical circuitry either through direct connections or indirect poly-synaptic connections [24], there is substantial evidence that weighted usage of the edge varies according to cognitive contexts [25], [26]. In other words, different cognitions may share an anatomical edge, but may differ in weighted usage of that edge. The weight for a particular edge may be associated with various factors such as the number of fibers connecting the two regions, firing rates, and modulation by other neurons that dynamically changes according to the neural context [3]. Graph-ICA has the capacity to decompose the weighted usage of each edge, which composes an independent subnetwork. This differs from previous subnetwork decomposition methods, including decomposing exclusive nodes and edges using modularity optimization [20] or shared nodes and edges but with identical edge strengths (across multiple subnetworks) using spatially-overlapping graph clustering methods [27]–[30]. In graph-ICA, multiple subnetworks can share same edges but with different edge strengths.

Various cognitive functions may recruit different sets of spatially distributed components with different strengths [31], [32]. In that respect, encoding brain states (or cognitions) can be understood in terms of context-dependent recruitment and release of a set of functional subnetworks. This hypothesis was tested with motor and cognitive tasks, which increased usage-strengths of the subnetwork components corresponding to the task. For example, during a motor task, a usage-strength increase was found within a motor-related subnetwork based in the precentral cortex (IC19), similar to the results reported by Damoiseaux *et al*.[33]. During a working memory task, the fronto-parietal subnetwork (IC35) and subnetwork based in Broca’s region (IC37) were highly active. The verb-generation task increased usage-strength in the subnetwork corresponding to the inferior frontal gyrus, which was consistent with previously reported results [34]. The n-back task elicited increased strength of the fronto-parietal subnetwork, which corresponds to well-known working memory circuits including the dorsolateral prefrontal cortex, inferior frontal gyrus, and parietal lobe [35]. These examples suggest that cognitive processing can be modeled with mixtures of independent subnetwork components.

It is noteworthy that the functional subnetworks involved in cognition and brain states are derived from networks in the resting state. It has been suggested that networks involved in cognition are a subset of networks embedded in spontaneous activity [31], [32], [36]. Considerable correspondence was found between resting state subnetworks and task-evoked activation/deactivation patterns in diverse cognitive imaging studies [32]. Thus, sensory tasks likely activate intrinsic subnetworks embedded in spontaneous activity rather than compose a new subnetwork for a given task [37], [38]. It is possible that ongoing rehearsals or recirculation of subnetworks involved with cognition [39] otherwise remain weak during the resting state.

Individual variability in human brain networks [40] is a well-known phenomenon that helps to explain variations in behavior and cognition. In the current study, independent subnetworks were extracted based on individual variability of connections between two nodes, which are the basis of a subnetwork.

The variability in usage-strength for a subnetwork is not limited to individuals, as it is seen with respect to sex as well. For example, the usage-strengths of subnetworks differed between males and females in the network based in the PCC/precuneus (IC34), in the network connecting the medial prefrontal and limbic regions (IC3), and in the network based in the middle cingulate cortex (IC28). In previous studies, the spatial pattern of the default mode network did not show a sex difference [41], but the density of functional connections was larger in females than in males [42]. In the current study, females showed increased strength in the anterior default mode subnetwork (IC3), but decreased strength in the posterior default mode subnetwork (IC34) compared to males during the resting state. Aside from these specific subnetworks, most others were consistently used independent of sex. Thus, the use of graph-ICA over the modularity optimization method [20] is advantageous in that it provides a measure for group-level comparisons of subnetworks.

The concept of deriving independent subnetwork components from cross-sectional data is based on two main assumptions: the existence of general subnetworks that are common across individuals, and the existence of variations across individuals in utilizing general subnetworks. This approach is similar to previous ICA applications to cross-sectional neuroimaging data that were used to find intrinsic independent sources in human brains. These studies sought to find task-specific cognitive components using cross-sectional perfusion PET data [43], to compare networks in the resting state fMRI with cross-sectional metabolic PET data [44], and to identify group specific components of gray matter density [45]. The current method is the first to apply ICA to graphs. Compared to voxel-level spatial ICA that is focused on weighted activity at each node [46], graph-ICA focuses on weighted associations among nodes. Although graph-ICA utilizes adjacency matrices of individuals in contrast to spatial ICA that uses spatio-temporal data, graph-ICA has capacity to identify functional subnetworks found in spatial ICA. When compared with the conventional modularity optimization method [20], we obtained weighted and shared associations between nodes rather than fixed associations.

In summary, graph-ICA has several advantages over previous methods. First, graph-ICA decomposes subnetworks with spatially-overlapping and weighted edges, which is not supported in the modularity optimization [47] and a graph clustering method with spatially-overlapping but fixed edges [29]. Second, graph-ICA provides more direct information on the edge weights, i.e., connectivity, among brain regions working together as a network, which cannot be directly obtained through voxel-level spatial ICA. In spatial ICA, the connectivity strengths among clusters within a component are not clearly defined. Third, graph-ICA facilitates a group-level comparison of subnetwork usages by comparing individualized weights for each subnetwork. This is not supported in conventional modularity optimization or other approaches.

Graph-ICA may be dependent on the node definition to construct a graph. The definition of nodes used in our study is based on anatomically defined maps that may not correspond to functional nodes. Functional connectivity derived from functionally homogeneous nodes [48], [49] would provide more reliable results for graph-ICA.

The application of graph-ICA has several challenges, similar to conventional ICA, such as determination of number of graph sources and rejection of artifactual components. Also, efficient visualization techniques of overlapping subgraphs are essentially needed. All these challenges wait for further research.

In evaluating task-specific subnetworks using n-back task, we calculated adjacency matrices from different durations (sample sizes) of 0-back (25s) and 2-back (30s) task blocks, which might affect functional connectivity estimation due to different degrees of freedom. Although this may not be critical in the current study, more reliable evaluation may require same sample sizes across conditions. In the graph-ICA, we assumed a set of common subnetwork components but variable usage strengths of those components according to subjects, tasks and sex groups. This study can be further extended to allow group-specfic repertoires of subnetworks and their usage strengths, which may be more efficient in characterizing individuals, groups and brain functions.

In the current study, graph-ICA is limited in explaining bidirectional associations, as the adjacency matrix used in this study contains no directional information. If we could derive brain networks using bidirectional models such as dynamic causal modeling [4], graph-ICA could be used to decompose common brain network constructs based on directional connectivity. The subnetworks identified using graph-ICA may be regarded as basic models common to all individuals onto which sophisticated functions may be constructed. Thus, more sophisticated network modeling may be constructed on these data-driven subnetworks.

## Conclusions

Our results suggest that functional subnetwork repertoires can be decomposed using independent component analysis based on a small number of cross-sectional whole-brain networks. Our simulation and cognitive task studies further suggest that this method can effectively be utilized to identify task-specific functional subnetworks both in individual and in group data.

## Supporting Information

Figure S1 Simulation results of graph-ICA with different contrast-to-noise ratios (CNR) from 0.5 to 2.
(JPG)

Figure S2 Functional subnetworks estimated by graph-ICA (continued on next page). Brain local regions (nodes) and edges were color-coded to mixture of red, green, and blue for suitable identification.
(JPG)

Text S1 Modularity optimization
(PDF)
