# Supplementary figures and images for: Graph Independent Component Analysis Reveals Repertoires of Intrinsic Network Components in the Human Brain
Source: PLoS One. 2014 Jan 7;9(1):e82873. doi: 10.1371/journal.pone.0082873 (PMC3883640; doi:10.1371/journal.pone.0082873)

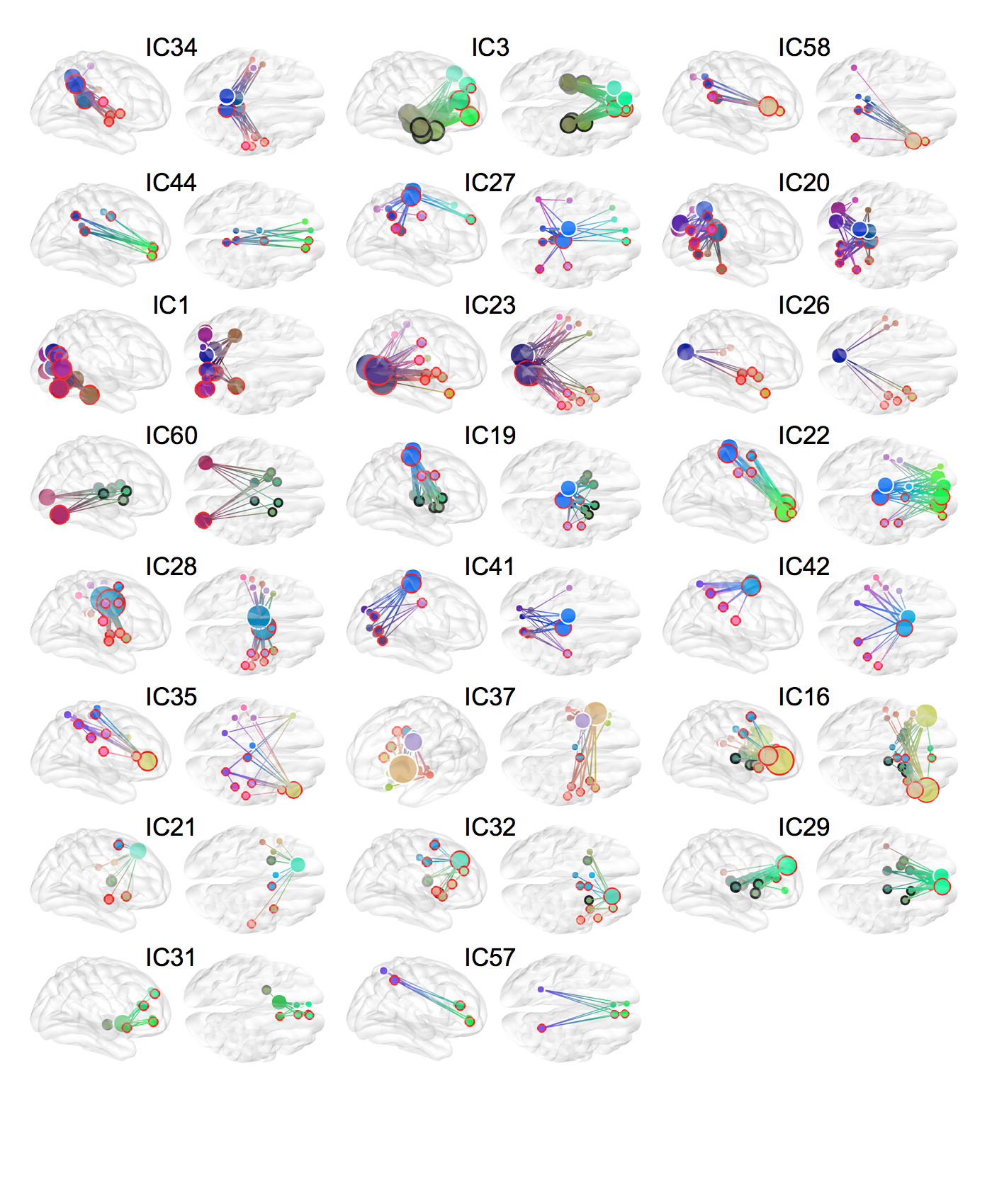

Supplement: Figure S1 — Simulation results of graph-ICA with different contrast-to-noise ratios (CNR) from 0.5 to 2. (JPG) [file pone.0082873.s001.jpg]

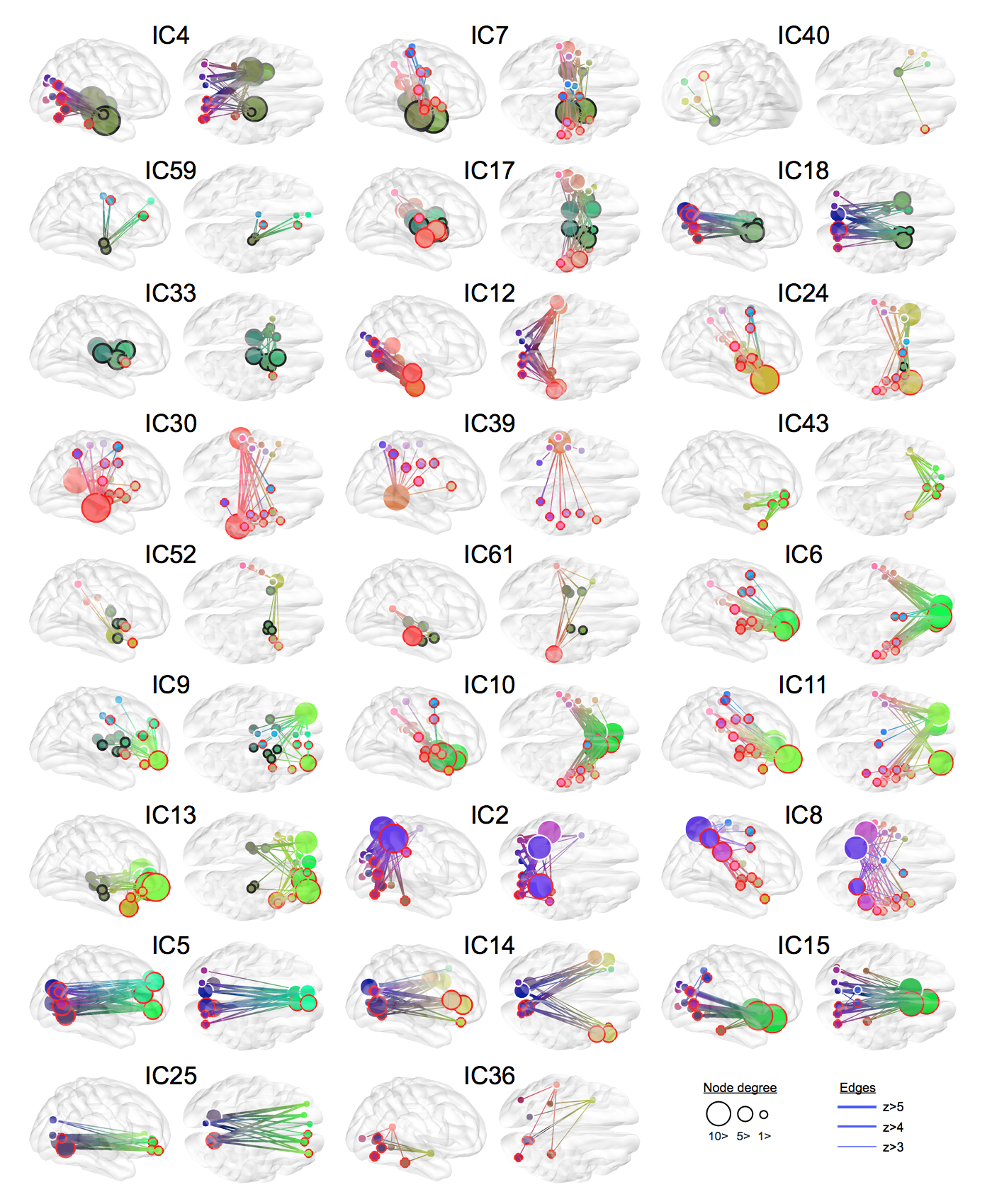

Supplement: Figure S2 — Functional subnetworks estimated by graph-ICA (continued on next page). Brain local regions (nodes) and edges were color-coded to mixture of red, green, and blue for suitable identification. (JPG) [file pone.0082873.s002.jpg]
