# Supplementary material for: Graph Independent Component Analysis Reveals Repertoires of Intrinsic Network Components in the Human Brain
Source: PLoS One. 2014 Jan 7;9(1):e82873. doi: 10.1371/journal.pone.0082873 (PMC3883640; doi:10.1371/journal.pone.0082873)
Supplement: Text S1 — Modularity optimization (PDF) [file pone.0082873.s003.pdf]

Text S1. Modularity optimization

Modularity (Q) is defined as (total connection weight within the subnetwork) – (chance-expected total connection weight) and modularity optimization is to find network-partitioning that maximizes Q [1]. This mathematical concept is expressed as follows:

$$Q = \frac{1}{V}\sum_{ij}\left(w_{ij} - e_{ij}\right)\delta_{M_i M_j} \quad (1)$$

$$e_{ij} = \frac{s_i s_j}{V},\ s_i = \sum_j w_{ij},\ V = \sum_{ij} w_{ij} \quad (2)$$

where e_{ij} is the chance-expected total weight, w_{ij} is the connection weight between node i and j, V is the total sum of weights to which Q is rescaled [0,1], and $\delta_{M_i M_j}$ is an indicator with a value of 1 if node i and j are in the same subnetwork and a 0 value, otherwise.

To compare with subnetworks identified by graph-ICA, we performed weighted modularity optimization. Prior to modularity optimization, we conducted Fisher's r-to-z transformations of inter-regional functional connectivity among 90 cortical regions for each subject and averaged them. Resulting modules (subnetworks) were normalized z-scores in spatial dimension with a threshold of z>3.

Reference

1. Newman ME (2006) Finding community structure in networks using the eigenvectors of matrices. *Phys Rev E Stat Nonlin Soft Matter Phys* 74(3 Pt 2):036104.
